# Supplementary material for: Mechanochemically responsive polymer enables shockwave visualization
Source: Nat Commun. 2024 Oct 7;15:8596. doi: 10.1038/s41467-024-52663-1 (PMC11458618; doi:10.1038/s41467-024-52663-1)
Supplement: Supplementary file 1 — Supplementary Information [file 41467_2024_52663_MOESM1_ESM.pdf]

Supplementary Information:
Mechanochemically Responsive Polymer Enables Shockwave Visualization

Polette J. Centellas[1], Kyle D. Mehringer[2], Andrew L. Bowman[3], Katherine M. Evans[1], Parth Vagholkar[2], Travis L. Thornell[3], Liping Huang[4], Sarah E. Morgan[2], Christopher L. Soles[1], Yoan C. Simon[5*], Edwin P. Chan[1*]

[1]Materials Science and Engineering Division, National Institute of Standards and Technology, 100 Bureau Dr, Gaithersburg, 20899, MD, USA.
[2]School of Polymer Science and Engineering, University of Southern Mississippi, 118 College Dr, Hattiesburg, 39406, MS, USA.
[3]Geotechnical and Structures Laboratory, US Army Engineer Research and Development Center, 3909 Halls Ferry Rd, Vicksburg, 39180, MS, USA.
[4]Department of Materials Science and Engineering, Rensselaer Polytechnic Institute, 110 8th St, Troy, 12180, NY, USA.
[5]School of Molecular Sciences, Arizona State University, Physical Sciences Center PSD 104, Tempe, 85287, AZ, USA.

*Corresponding author(s). E-mail(s): yoan.simon@asu.edu; edwin.chan@nist.gov;
Contributing authors: polette.centellas@nist.gov; kyle.mehringer@usm.edu; Andrew.L.Bowman@erdc.dren.mil; katherine.evans@nist.gov; Parth.Vagholkar@usm.edu; Travis.L.Thornell@erdc.dren.mil; huangL5@rpi.edu; sarah.morgan@usm.edu; christopher.soles@nist.gov;

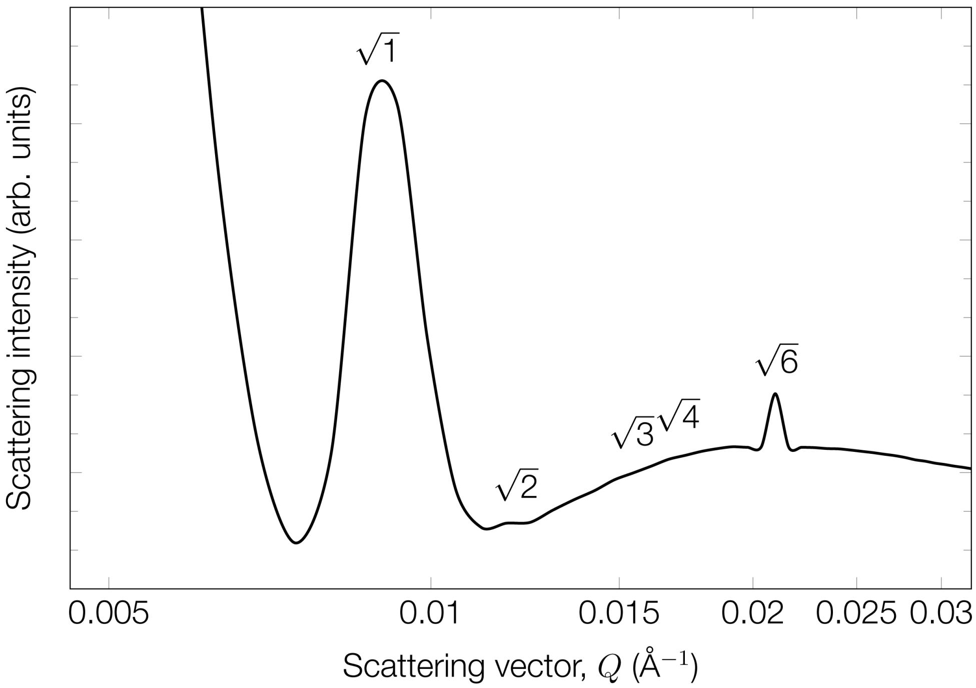

Supplementary Fig. 1 Small-angle X-ray scattering (SAXS). SAXS data for the mechanophore-functionalized block copolymer with background subtraction. The ratio of the scattering peaks $\sqrt{1} : \sqrt{2} : \sqrt{3} : \sqrt{4} : \sqrt{6}$: indicates a spherical morphology.

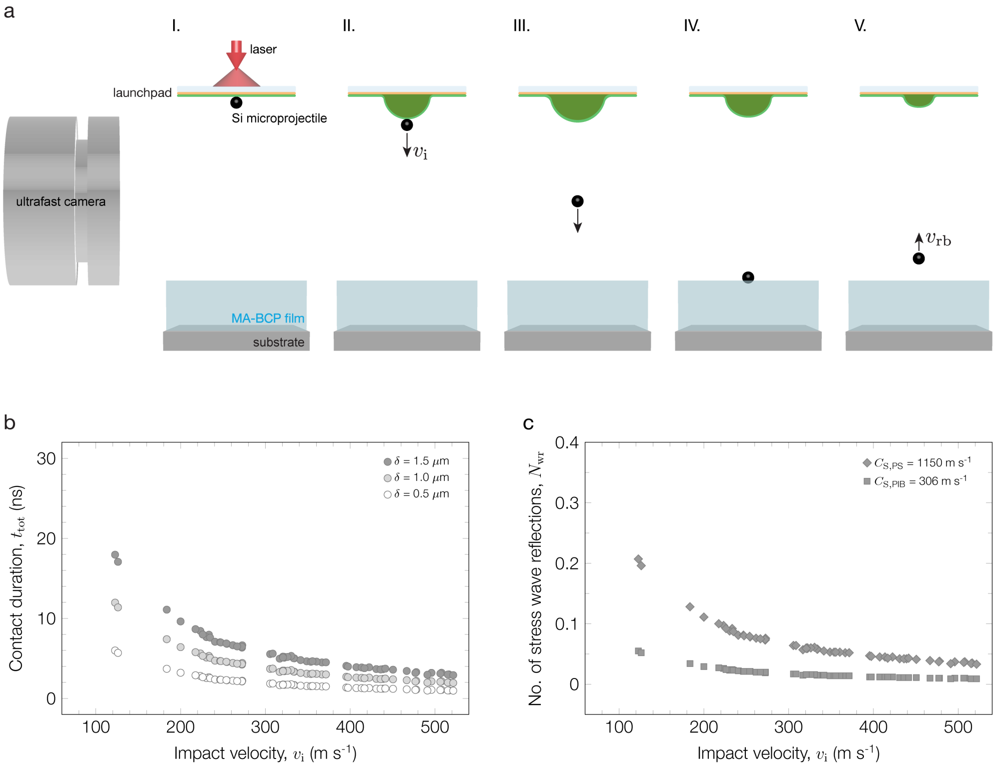

Supplementary Fig. 2 Microballistic impact experiments. a, Schematic of the LIPIT platform used to perform microballistic impact tests. **I-V,** Temporal evolution of an impact test. **b,** Calculated contact time between the microprojectile and MA-BCP film (t_{tot}) assuming a range of contact depths (δ) for the range of impact velocities (v_{i}) tested. **c,** Estimated number of stress wave reflections (N_{wr}) across the MA-BCP film thickness ($\approx 50\ um$) assuming a contact depth of $\delta = 1.5$ um. No stress wave reflections ($N_{\mathrm{wr}} < 1$) are predicted for the shear wave velocity of the PS block ($C_{\mathrm{S,PS}}$) nor the shear wave velocity of the PIB block ($C_{\mathrm{S,PIB}}$ extracted from FM measurements) at any v_{i} tested.

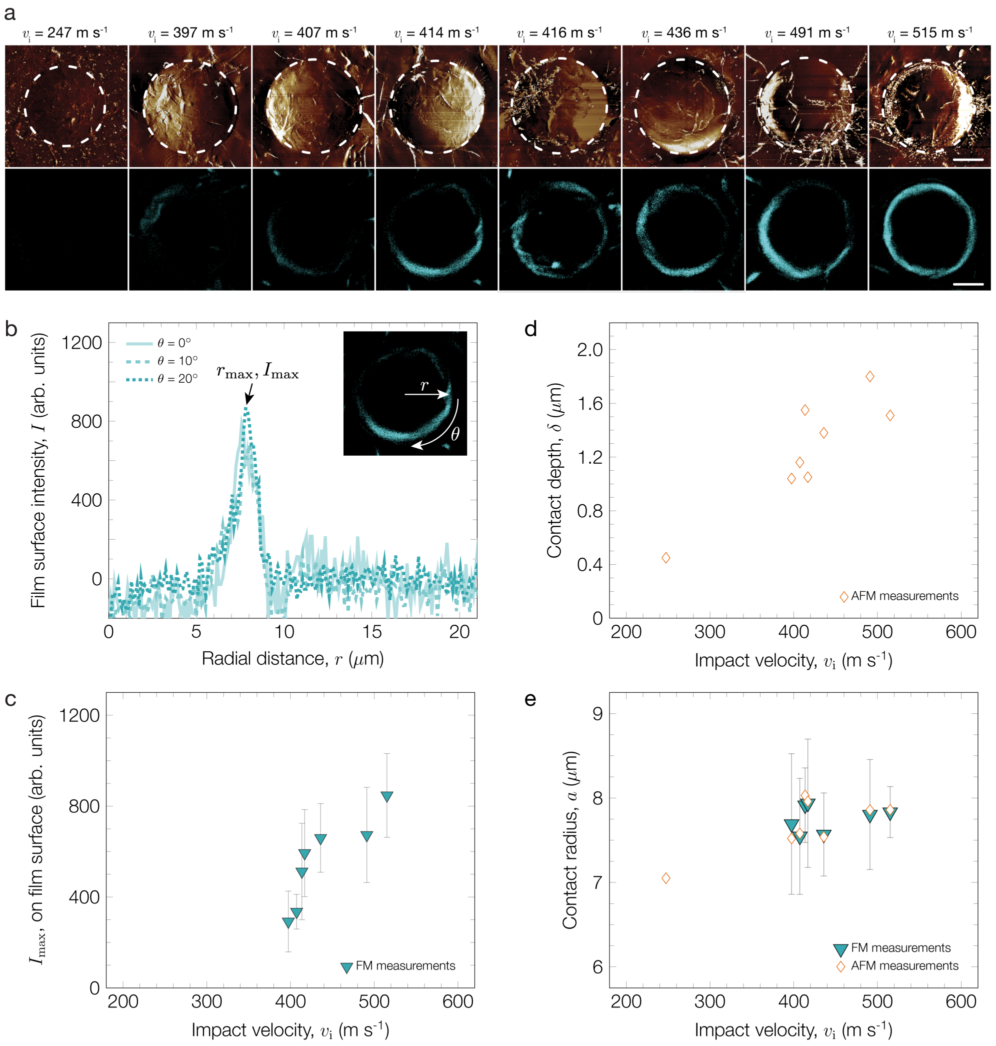

Supplementary Fig. 3 Surface characterization of impacted sites. a, AFM phase images (top row) and corresponding FM images (bottom row) for eight different impacted sites. Scale bars represent 5 μm. **b,** Representative radial intensity profiles at $\theta = 0°$, $10°$, and $20°$ taken on the film surface ($z = 0$ plane) for an intersonic impact case ($v_\mathrm{i} = 414$ m s^{-1}). The maximum fluorescence intensity and corresponding radial location are defined as I_max and r_max, respectively, for each profile. **c** Averaged I_max, measured by FM, and **d,** contact depth (δ) of the impacted site, measured by AFM, for varying impact velocities (v_i). Data are presented as mean values $\pm$ SD ($n = 36$). **e,** Comparison of contact radius (a) of impacted sites measured by AFM and FM. Data are presented as mean values $\pm$ SD ($n = 36$). In **c** and **e** there is no detectable fluorescence signal measured for the $v_\mathrm{i} = 247$ m s^{-1} (i.e., subsonic impact) case.

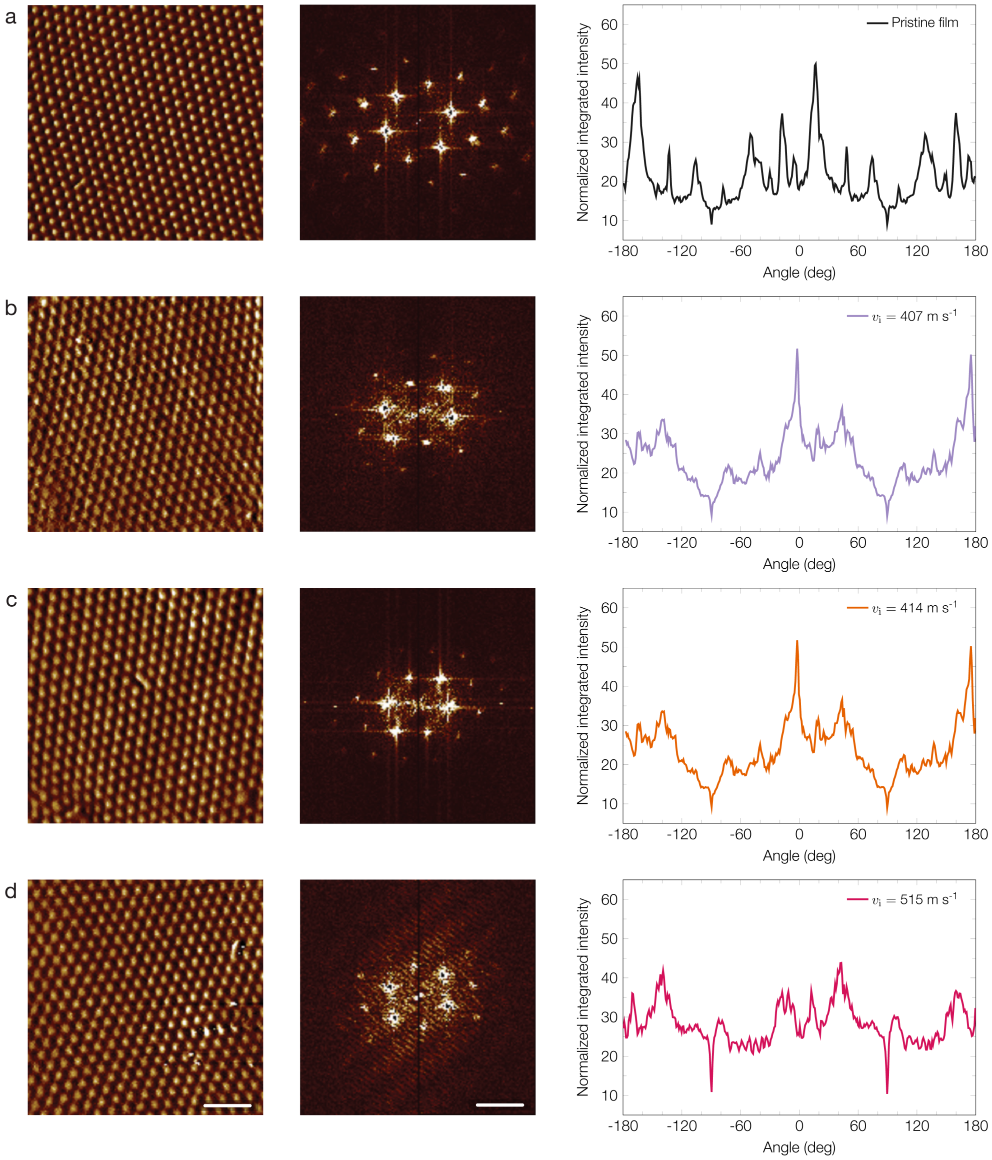

Supplementary Fig. 4 Characterization of MA-BCP periodicity and ordering. AFM phase image (left), FFT of AFM phase image (middle) and azimuthal integration on FFT (right) corresponding to the **a,** pristine (undamaged) film, **b,** $v_{\mathrm{i}} = 407$ m s^{-1} impacted site, **c,** $v_{\mathrm{i}} = 414$ m s^{-1} impacted site and **d,** $v_{\mathrm{i}} = 515$ m s^{-1} impacted site. Scale bars represent 300 nm.

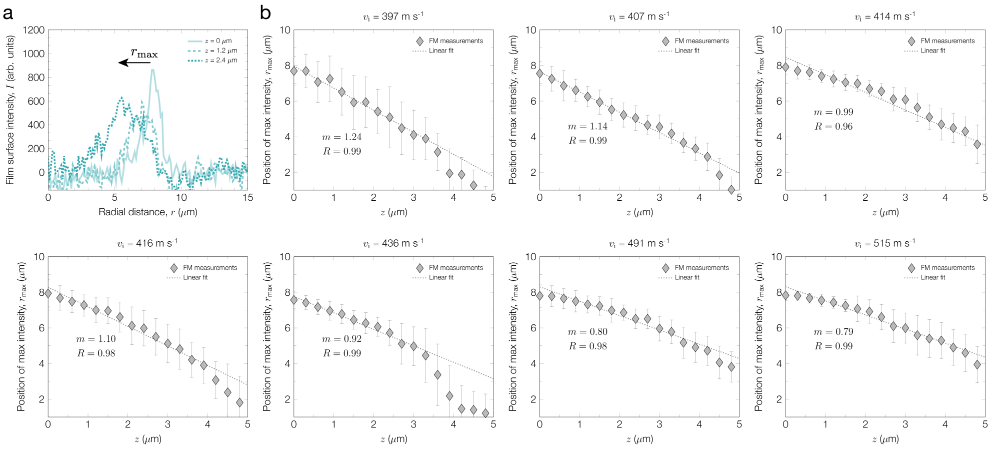

Supplementary Fig. 5 Calculation of cone angles from FM measurements. a, Representative radial intensity profiles for selected depths of $z = 0$ μm, 1.2 μm, and 2.4 μm for an intersonic impact case ($v_\mathrm{i} = 414$ m s^{-1}). **b,** Subsurface mechanophore activation is observed for seven different impacted sites from $v_\mathrm{i} = 397$ m s^{-1} to 515 m s^{-1}. The cone angle (α) of the mechanophore-activated volume is extracted from the slope (m) of the best-fit trendline for r_max versus z according to $\alpha = \tan^{-1}(m)$. Data are presented as mean values ± SD ($n = 36$).

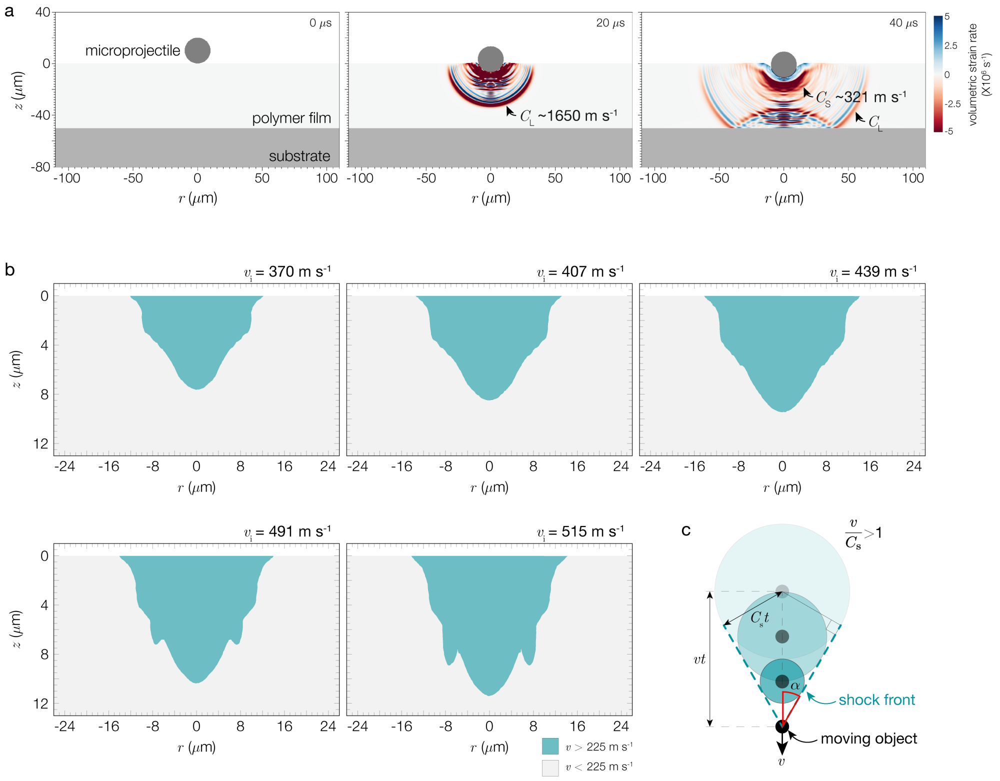

Supplementary Fig. 6 FEA results. a, Volumetric strain rate maps showing the propagation of pressure (C_{L}) and shear waves (C_{S}) into the polymer film as the projectile impacts the target. **b,** Peak nodal velocity maps as a function of projectile impact velocity. **c,** Schematic of shear Mach cone formation during impact event.

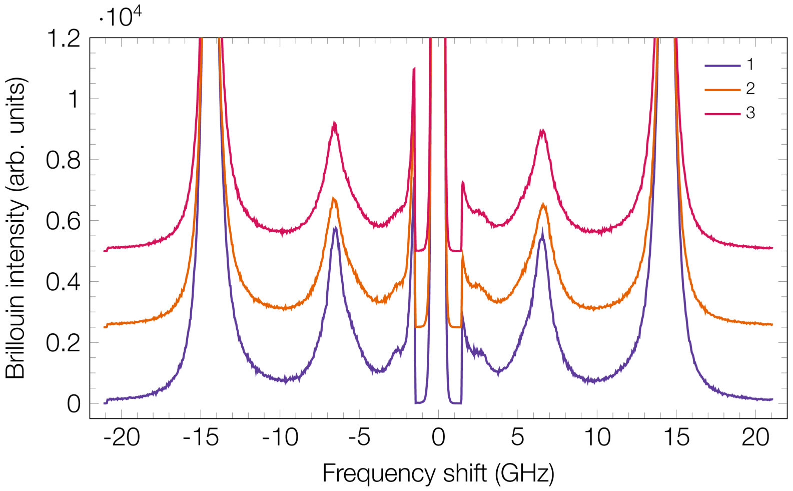

Supplementary Fig. 7 BLS results. The three curves correspond to three separate BLS measurements of the MA-BCP.

Supplementary Table 1 AFM measurements, fluorescence microscopy measurements, and penetration strain rate calculations for varying impact velocities.

v_i (m s^{-1})	δ (μm)	a (μm)	r_{max}[1] (μm)	Error[2] (%)	$\dot{\varepsilon} \times 10^8$ (s^{-1})
247	0.45	7.05	N/A	N/A	5.5
397	1.04	7.52	7.69 ± 0.8	2.3	3.8
407	1.16	7.58	7.55 ± 0.7	0.5	3.5
414	1.55	8.03	7.91 ± 0.4	1.5	2.7
417	1.05	7.96	7.94 ± 0.8	0.4	4.0
436	1.38	7.54	7.57 ± 0.5	0.4	3.2
491	1.80	7.86	7.80 ± 0.7	0.7	2.7
515	1.51	7.86	7.83 ± 0.3	0.3	3.4

[1]Fluorescence measurement corresponds to the film surface.

[2]Calculated for contact radius measured by AFM (a) compared to fluorescence microscopy (r_{max}) according to $\left(\frac{a-r_{max}}{a}\right) \times 100$.

Supplementary Methods

The detailed synthesis and characterization was reported in our publication titled 'Synthesis and Morphology of High-Molecular-Weight Polyisobutylene-Polystyrene Block Copolymers Containing Dynamic Covalent Bonds'. Following is the synthetic procedure used to make the diblock copolymer:

Synthesis of monofunctional bromine terminated polyisobutylene (PIB-Br)

Bromine terminated polyisobutylene (PIB-Br) was synthesized in a two-step process with the polymerization of isobutylene followed by its quenching with alkoxybenzene. In the first step, isobutylene (IB, 710 mmol, 57 mL) was polymerized in a 500 mL round bottom flask (equipped with a mechanical stirrer) within a drybox via quasi-living cationic polymerization at -60 °C using 2-chloro-2,4,4-trimethylpentane (TMPCl, 0.5 mmol, 0.08 mL) as the initiator, titanium tetrachloride ($TiCl_4$, 11.3 mmol, 1.24 mL) as the catalyst, and 2,6-lutidine (0.7 mmol, 0.09 mL) as the Lewis base in a 60/40 (v/v) solvent system containing hexane (106.91 mL)/methyl chloride (71.27 mL). FTIR (ReactIR 4000) was used to monitor isobutylene by observing the olefinic $=CH_2$ wag of IB (887 cm^{-1}). Upon full conversion of IB (complete disappearance of the olefinic wag peak), 3-bromopropoxy)benzene (10 mmol, 1.57 mL) was added, and the reaction was brought to -72 °C. $TiCl_4$ (22.6 mmol, 2 mL) was then injected into the flask, and the reaction was stirred overnight until termination by the addition of excess methanol. The resulting solution was warmed to room temperature and then precipitated into methanol. The precipitate was collected, dissolved in hexanes, and reprecipitated in methanol. The precipitate was dissolved in hexanes and washed twice with deionized water followed by a saturated sodium chloride solution, dried over $MgSO_4$, filtered, and vacuum stripped to yield the isolated polymer (37 g, M_n = 5.95 x 10^4 g/mol , M_w/M_n = 1.04, 94% yield).

Synthesis of monofunctional PIB oxanorbornene (PIB-MI)

PIB-Br (15 g, 0.25 mmol) was dissolved in THF (200 mL) and DMF (40 mL). exo-7-oxanorbornene-2,3-dicarboximide (1.14 g, 6.95 mmol), K_2CO_3 (1.19 g, 8.57 mmol) and 18-crown-6 (0.93 g, 3.5 mmol) were added to the stirring solution. The mixture was heated at 50 °C for 12 h under nitrogen atmosphere. Upon completion, the THF was removed by rotary evaporation, and the remaining product was dissolved in hexanes, filtered, and precipitated by slowly adding into a large excess of methanol. The polymer was collected, dissolved in hexanes, washed 3x with distilled water, dried over $MgSO_4$, and vacuum stripped at room temperature to obtain the final product (11.2 g, M_n = 5.4 x 10^4 g/mol, M_w/M_n = 1.04, 75% yield).

Synthesis of (2-(((dodecylthio)carbonothioyl)thio)-2-methylpropanoic acid) DMP

Acetone (180 mL) and K_3PO_4 (12.78 g, 59.3 mmol) were added to a 500 mL flame-dried round bottom flask and put under N_2 atmosphere. Dodecane thiol (14.2 mL, 59.3 mmol) was added to the solution over 15 min using an addition funnel, and the mixture was allowed to stir for an additional 5 min. CS_2 (9.67 mL, 160.1 mmol) was then added dropwise, causing the solution to turn a yellow-orange color. After stirring for 1 h, 2-bromo-2-methylpropionic acid (8.82 g, 52.8 mmol) was added to the flask, and the reaction was allowed to stir overnight. The next day, the mixture had solidified to a solid yellow paste. The paste was dissolved with EtOAc and washed with HCl (1 M) followed by a brine wash. The organic phase was dried overnight with $MgSO_4$ which was subsequently removed by filtration. The filtrate was concentrated via rotary evaporation and then purified via gradient column chromatography (5→10% EtOAc in hexanes) to yield a yellow crystalline solid (16.6 g, 86.2% yield).
^{1}H NMR (600 MHz, Chloroform-*d*) δ 3.30 (t, J = 3.3 Hz, 1H), 1.74 (s, 6H), 1.69 (p, J = 7.5 Hz, 2H), 1.40 (p, J = 7.0 Hz, 3H), 1.35 – 1.23 (m, 16H), 0.89 (t, J = 7.0 Hz, 3H). ^{13}C NMR (151 MHz, $CDCl_3$) δ 178.71 55.58, 37.10, 31.93, 29.65, 29.65, 29.64, 29.57, 29.47, 29.36, 29.13, 28.99, 27.84, 25.24, 22.70, 14.12.

Synthesis of anthracen-9-ylmethyl DMP (AMDMP)

DMP (16.55 g, 45.4 mmol), EDC·HCl (13.05 g, 68.1 mmol), DMAP (8.32 g, 68.1 mmol), and DCM (100 mL) were combined in an oven-dried 500 mL 3 neck round bottom flask to yield a heterogeneous orange solution with a white solid. A solution of 9-anthracenemethanol (14.23 g, 68.1 mmol) in DCM (50 mL) was added to the flask, and the mixture was stirred overnight under N_2. The reddish-brown solution was washed with $NaHCO_3$ (1 M), followed by HCl (1 M), and finally with a brine wash. After the washes, the dark red solution was dried with $MgSO_4$ and filtered to remove any solids. The filtrate was concentrated via rotary evaporation and purified via column chromatography (6% EtOAc in hexanes) yielding a yellow crystalline solid (14.7 g, 58.6% yield).
^{1}H NMR (600 MHz, Chloroform-*d*) δ 8.50 (s, 1H), 8.34 (d, J = 8.9 Hz, 2H), 8.03 (d, J = 8.4 Hz, 2H), 7.59 – 7.46 (m, 4H), 6.16 (s, 2H), 3.17 (t, J = 8.1, 6.8 Hz, 2H), 1.67 (s, 6H), 1.58 (p, J = 7.3 Hz, 2H), 1.29 (s, 18H), 0.89 (t, J = 7.0 Hz, 3H).

General procedure for the RAFT polymerization of polystyrene (PS-Anth)

To an oven dried 500 mL Schlenk flask was added: AMDMP (0.53 g, 0.96 mmol), AIBN (0.02 g, 0.09 mmol), styrene (115.2 g, 1106 mmol), and toluene (80 mL). The solution was subjected to 3 freeze-pump-thaw cycles to remove oxygen. The flask was backfilled with argon and warmed with stirring to 70 °C. Aliquots were periodically removed under argon for NMR spectroscopy. Upon reaching ca. 50% conversion, the reaction was removed from heat, quenched in liquid N_2, and opened to air. The polymer was

purified by precipitation into MeOH, and reprecipitation from THF was performed to ensure purity. The resulting solid polymer (34 g, M_n = 5.7 x 10^4 g/mol, M_w/M_n = 1.13, 58% yield) had a yellow tint from the trithiocarbonate of the RAFT agent.

General procedure for preparation of diblock PIB-PS copolymer via Diels-Alder reaction of PIB-MI and PS-Anth

PIB-MI (1.0 equiv) and PS-Anth (1.3 equiv) were dissolved in a minimal amount of toluene with a catalytic amount of BHT as a radical inhibitor. The reaction was sparged for 30 min with dry N_2 and refluxed in the dark. The DA reaction was monitored through ^{1}H NMR spectroscopy by the disappearance of the aliphatic protons at 3.86 and 3.62 ppm and the appearance of the bridgehead proton at 3.11, 3.24, and 4.72 ppm for ca. 6 days, or until the aliphatic protons of the (3-bromopropoxy)benzene at 3.62 and 3.86 ppm were no longer visible by ^{1}H NMR spectroscopy. Exo-7-oxanorbornene-2,3-dicarboximide (0.5 equiv) was then added to the solution and reflux was continued for 12 h. The solution was precipitated in acetone and centrifuged discarding the supernatant. The precipitate was resuspended in fresh acetone and centrifuged discarding the supernatant twice. Then the polymer was dispersed in a 12:1 pentane:MeOH (v:v) solvent mixture and centrifuged discarding the final supernatant three times. The polymer was dissolved in THF, precipitated in MeOH, vacuum filtered, and dried at 50 °C under high vacuum to obtain the final polymer.
